# Molecular cytogenetic characterization and phylogenetic analysis of four *Miscanthus* species (Poaceae)

**DOI:** 10.3897/CompCytogen.v13i3.35346

**Published:** 2019-08-09

**Authors:** Yan-Mei Tang, Liang Xiao, Yasir Iqbal, Jian-Feng Liao, Long-Qian Xiao, Zi-Li Yi, Chao-Wen She

**Affiliations:** 1 College of Bioscience and Biotechnology, Hunan Agricultural University, Changsha, Hunan 410128, China Hunan Agricultural University Changsha China; 2 Key Laboratory of Research and Utilization of Ethnomedicinal Plant Resources of Hunan Province, Huaihua University, Huaihua, Hunan 418008, China Huaihua University Huaihua China

**Keywords:** *
Miscanthus
*, karyotype, fluorochrome banding, 45S ribosomal RNA genes (45S rDNA), *in situ* hybridization, internal transcribed spacers, phylogeny

## Abstract

Chromosomes of four *Miscanthus* (Andersson, 1855) species including *M. sinensis* (Andersson, 1855), *M. floridulus* (Schumann & Lauterb, 1901), *M. sacchariflorus* (Hackel, 1882) and *M. lutarioriparius* (Chen & Renvoize, 2005) were analyzed using sequentially combined PI and DAPI (CPD) staining and fluorescence *in situ* hybridization (FISH) with 45S rDNA probe. To elucidate the phylogenetic relationship among the four *Miscanthus* species, the homology of repetitive sequences among the four species was analyzed by comparative genomic *in situ* hybridization (cGISH). Subsequently four *Miscanthus* species were clustered based on the internal transcribed spacer (ITS) of 45S rDNA. Molecular cytogenetic karyotypes of the four *Miscanthus* species were established for the first time using chromosome measurements, fluorochrome bands and 45S rDNA FISH signals, which will provide a cytogenetic tool for the identification of these four species. All the four have the karyotype formula of *Miscanthus* species, which is 2n = 2x = 38 = 34m(2SAT) + 4sm, and one pair of 45S rDNA sites. The latter were shown as strong red bands by CPD staining. A non-rDNA CPD band emerged in *M. floridulus* and some blue DAPI bands appeared in *M. sinensis* and *M. floridulus*. The hybridization signals of *M. floridulus* genomic DNA to the chromosomes of *M. sinensis* and *M. lutarioriparius* genomic DNA to the chromosomes of *M. sacchariflorus* were stronger and more evenly distributed than other combinations. Molecular phylogenetic trees showed that *M. sinensis* and *M. floridulus* were closest relatives, and *M. sacchariflorus* and *M. lutarioriparius* were also closely related. These findings were consistent with the phylogenetic relationships inferred from the cGISH patterns.

## Introduction

The genus *Miscanthus* (Andersson, 1855), belonging to the tribe Andropogoneae of family Poaceae, is a tall perennial grass with C4 photosynthesis (Stewart et al. 2009). It includes 14–20 species and has been considered as one of the most promising high-yield fiber-based energy crops (Christian et al. 2009, Brosse et al. 2012). China is a genetic center of diverse *Miscanthus* germplasm. Four *Miscanthus* species, *M. sinensis* (Andersson, 1855), *M. floridulus* (Schumann & Lauterb, 1901), *M. sacchariflorus* (Hackel, 1882) and *M. lutarioriparius* (Chen & Renvoize, 2005), are most widely distributed. These have high biomass yield and are prone to interspecific hybridization, which lead to high genetic diversity (Liu et al. 2013, Zhao et al. 2017).

*M. sinensis* has been already sequenced (*Miscanthus sinensis* v7.1 DOE-JGI, https://phytozome.jgi.doe.gov/) and is an important species for comparative genomics. Therefore, it is necessary to investigate the chromosomes of *M. sinensis* and other *Miscanthus* species and reveal their genomic homology. It will provide a reference for further development of specific probes based on the *M. sinensis* genome sequence for chromosomal localization in *Miscanthus* and related genera. *M. floridulus* is similar to *M. sinensis* in morphology. *M. lutarioriparius* is a native *Miscanthus* species of China (Chen and Renvoize 2006, Sheng et al. 2016, Yang et al. 2019). Some scholars have published it as a variant or subspecies of *M. sacchariflorus* (Liu 2009, Sun et al. 2010, Lu 2012, Hu 2015) because of their high similarity in morphology. In a word, the interspecific relationships of the four *Miscanthus* species are complex and their origins are unclear (Chen and Renvoize 2006, Tamura et al. 2016). Until now, taxonomic studies on *M. sinensis*, *M. floridulus*, *M. sacchariflorus* and *M. lutarioriparius* have been carried out by using morphological features (Chae et al. 2014, Yook et al. 2014), nuclear DNA content (Chae et al. 2014, Sheng et al. 2016), molecular markers (Chae et al. 2014, Yook et al. 2014, Tang et al. 2015), fluorescence *in situ* hybridization (FISH) (Takahashi and Shibata 2002, Takahashi et al. 2002), and spectroscopy (Jin et al. 2017). However, none of them could distinguish the four species from each other unequivocally. Karyotype, the characterization of a genome at the chromosomal level, is a valuable tool for species identification and evolution analysis (Silva et al. 2018). However, it is difficult to perform accurate karyotype analysis in *Miscanthus* species because of their little chromosomal differentiation and lack of distinct chromosomal landmarks. Chromosome banding by Giemsa staining, fluorochrome staining or FISH with repetitive DNA sequences can provide additional characteristics to discriminate the chromosomes in the cell complement (Filion 1974, Sumner 1990, Koornneef et al. 2003).

Fluorochrome banding techniques use fluorescent dyes preferentially binding to GC- or AT-rich DNA sequences to display different classes of heterochromatin on chromosomes (Sumner 1990). Among the techniques used, combined PI (propidium iodide) and DAPI (4’, 6 diamino-2-phenylindole dihydrochloride) staining (called CPD staining) can reveal simultaneously GC-rich and AT-rich regions along chromosomes with high precision and repeatability (Peterson et al. 1999, She et al. 2006, 2015, 2017). FISH with 5S and 45S rDNA probes have been widely applied in plants to determine the number and location of rDNA sites, and to provide effective markers for chromosome identification. Moreover, information on evolutionary relationships between species can be provided by comparing rDNA distribution characteristics between closely related species (Moscone et al. 1999, de Moraes et al. 2007, She et al. 2015, 2017). The combination of chromosome morphology, fluorochrome bands and FISH signals can be employed to construct molecular cytogenetic karyotype. It can reveal chromosome-level genome organization of a plant species, investigate the evolutionary relationships among related species, and integrate genetic and physical maps (Zhang et al. 2015, She et al. 2015, 2017). Cytogenetic studies in the four *Miscanthus* species were so far primarily restricted to chromosome counts and conventional karyotype descriptions (Chramiec-Głąbik et al. 2012, Chae et al. 2014). FISH has been applied in diploid *M. sinensis* and tetraploid *M. sacchariflorus*, but their molecular cytogenetic karyotypes have not been established as yet (Takahashi and Shibata 2002, Takahashi et al. 2002).

The internal transcribed spacer (ITS) regions of 45S rDNA have been used extensively for determining phylogenetic relationships at interspecific or intraspecific level because of its relatively high rate of mutation (Álvarez and Wendel 2003, Hao et al. 2004, Capua et al. 2017). Another direct method for examining genome relationships is comparative genomic *in situ* hybridization (cGISH), in which the labelled total genomic DNA of one species is hybridized to the chromosomes of another species without competitive DNA (Zoller et al. 2001). It generates hybridization signals in regions of conserved repetitive DNA sequences. Therefore, it can be used to identify the evolutionary relationships between species within a genus (Wolny and Hasterok 2009, She et al. 2015, Zhang et al. 2015). So far, the phylogenetic relationships among the four *Miscanthus* species were mainly carried out at the morphological, cellular and molecular levels (Chae et al. 2014, Yook et al. 2014). In previous studies, the ITS sequence was used to assess the phylogeny of the four *Miscanthus* species and the species of the *Saccharum* complex and other related genera (Hodkinson et al. 2002b, Chen et al. 2007, Liu et al. 2010), and the genome relationship between diploid *M. sinensis* and tetraploid *M. sacchariflorus* was examined by FISH with rDNA, genomic DNA and *Saccharum* centromeric repeats (Takahashi and Shibata 2002; Takahashi et al. 2002). However, there has been no study on the phylogenetic relationships of all the four species by combining molecular cytogenetic characterization with ITS sequence analysis.

In the current study, well spread mitotic metaphase chromosomes of four *Miscanthus* species were prepared using the modified flame-drying method. Chromosomes were characterized using sequential CPD staining and FISH with 45S rDNA probe. Detailed molecular cytogenetic karyotypes of these species were established using combined data of chromosome measurements, CPD bands, DAPI bands and 45S rDNA FISH signals. Meanwhile, cGISH was carried out to detect the homology of repetitive DNAs among these species, and a comparative sequence analysis of the ITS regions in these species was also conducted. The data were collected and evaluated to gain insight about the phylogenetic relationships among the four *Miscanthus* species.

## Material and methods

### Plant material and DNA extraction

Twenty-four *Miscanthus* accessions comprised of 6 *M. sinensis*, 6 *M. floridulus*, 6 *M. sacchariflorus* and 6 *M. lutarioriparius* were selected from different provinces of China and planted in the *Miscanthus* germplasm nursery located at the Hunan Agricultural University (Table 1). All the materials were used for ITS sequence analysis. Meanwhile, for CPD staining, rDNA FISH, karyotype analysis and cGISH, No. 03 (*M. sinensis*), No. 10 (*M. floridulus*), No. 16 (*M. sacchariflorus*) and No. 21 (*M. lutarioriparius*) were used. *Imperata cylindrica* (Beauvois, 1812) was included as an outgroup for the ITS phylogenetic analysis, and its sequences (JN407505.1) were obtained from GenBank Database.

**Table 1.** Geographical data of 24 Miscanthus accessions and GenBank Numbers of the ITS sequences

No.	Species	Orginal location	Longitude (E°)	Latitude (N°)	Altitude (m)	GenBank No.
01	*M. sinensis*	Huangshan, Anhui	118°15.78'E, 29°41.63'N		ca 139	MK981280
02		Shenzheng, Guangdong	114°18.00'E, 22°35.27'N		ca 27	MK981281
03		Wuhan, Hubei	104°24.46'E, 30°32.75'N		ca 735	MK138895
04		Jiaohe, Jilin	127°33.00'E, 43°34.00'N		ca 345	MK981282
05		Zibo, Shandong	117°50.11'E, 36°28.66'N		ca 290	MK981283
06		Naxi, Sichuan	105°27.43'E, 28°37.61'N		ca 400	MK981284
07	*M. floridulus*	Jinzhai, Anhui	115°43.30'E, 31°12.29'N		ca 490	MK981285
08		Nanpin, Fujian	110°17.36'E, 26°13.72'N		ca 97	MK981286
09		Qiongzhong, Hainan	109°54.03'E, 19°08.49'N		ca 263	MK981287
10		Wuhan, Hubei	114°24.46'E, 30°32.75'N		ca 45	MK138896
11		Wuzhou, Guangxi	111°22.35'E, 23°30.02'N		ca 25	MK981288
12		Zhuhai, Guangdong	113°35.99'E, 22°16.87'N		ca 2	MK981289
13	*M. sacchariflorus*	Jinzhai, Anhui	115°48.04'E, 31°12.29'N		ca 480	MK981290
14		Chengde, Hebei	117°50.50'E, 40°54.03'N		ca 351	MK981291
15		Ning’an, Heilongjiang	129°29.09'E, 44°23.84'N		ca 203	MK981292
16		Wuhan, Hubei	114°19.78'E, 30°28.60'N		ca 36	MK138897
17		Panshan, Liaoning	121°59.48'E, 41°14.57'N		ca 20	MK981293
18		Fuxian, Shaanxi	109°27.10'E, 35°59.30'N		ca 1246	MK981294
19	*M. lutarioriparius*	Tongling, Anhui	117°44.25'E, 30°51.69'N		ca 15	MK981295
20		Xichuan, Henan	111°28.69'E, 33°06.71'N		ca 168	MK981296
21		Wuhan, Hubei	114°19.52'E, 30°28.66'N		ca 78	MK138898
22		Changsha, Hunan	113°01.93'E, 28°11.08'N		ca 80	MK981297
23		Nanjing, Jiangsu	118°50.80'E, 32°04.37'N		ca 250	MK981298
24		Hukou, Jiangxi	116°12.68'E, 29°44.48'N		ca 9	MK981299

Total genomic DNA (gDNA) was extracted from fresh leaf tissue using the cetyltrimethylammonium bromide (CTAB) method described by Murray and Thompson (1980). The quality and concentration of DNA were measured by 1% agarose gel electrophoresis and a microplate reader (BioTek Instruments Inc, Winooski, USA).

### Chromosome preparation

Mitotic metaphase chromosomes were prepared by using the root tips according to the procedure described by She et al. (2006). The actively growing root tips were collected from potted plants and treated with saturated α-bromonaphthalene for 1.5 h at 28 °C, fixed in 3:1 (v/v) methanol/glacial acetic acid for at least 12 h at room temperature, and then stored at 4 °C until use. The fixed root tips were then washed in double distilled water and citrate buffer (0.01 mM citric acid-sodium citrate, pH 4.5) for 10 min each and incubated in a mixture of 2% cellulase R-10 (Yakult Pharmaceutical Industry, Tokyo, Japan), 2% pectolyase Y-23 (Yakult Pharmaceuticals), and 2% macerozyme R-10 (Sigma-Aldrich, Steinhem, Germany) in citric acid buffer at 28 °C for 2.5~3 h. Root tips were transferred to a glass slide along with the fixative and dissected using fine-pointed forceps. Finally, the slides were dried above a flame. Good preparations were selected by Olympus BX60 phase contrast microscope, and then stored at -20 °C.

### Staining with CPD and DAPI

The CPD staining followed the procedure described in She et al. (2006). Chromosome preparations were treated with RNase A and pepsin then stained with a mixture of 3 μg/ml DAPI and 0.6 μg/ml PI (both from Sigma-Aldrich) in a 30% (v/v, using double-distilled water as solvent) solution of Vectashield H-1000 (Vector Laboratories Burlingame, USA). Preparations were examined under an Olympus BX60 epifluorescence microscope equipped with a CoolSNAP EZ CCD camera (Photometrics, Tucson, USA). The CCD camera was controlled using Ocular software (Molecular Devices, Sunnyvale, USA). Photographs were taken using a green excitation filter for PI and a UV excitation filter for DAPI. DAPI and PI grey-scale images of the same plate were merged to produce a CPD image. The final images were optimized for contrast and background using Adobe PHOTOSHOP CS8.0.

### Probe DNA labeling

A 45S rDNA clone containing a 9.04-kb tomato 45S rDNA insert (Perry and Palukaitis 1990) were used as probe to localize the 18S-5.8S-26S ribosomal RNA gene. The DNAs (45S rDNA and gDNAs) of the four *Miscanthus* species were labeled with biotin-16-dUTP and digoxigenin-11-dUTP, respectively, using the Nick Translation Kit (Roche Diagnostics, Mannheim, Germany).

### FISH

FISH with the 45S rDNA probe was carried out on the same slides previously stained with CPD. FISH with the *M. sinensis*, *M. floridulus*, *M. sacchariflorus* and *M. lutarioriparius*genomic probes to the *M. sinensis* chromosomes. FISH with the *M. sacchariflorus*, *M. floridulus* and *M. lutarioriparius* genomic probes to the *M. sacchariflorus* chromosomes, and FISH with the *M. lutarioriparius* and *M. floridulus* genomic probes to the the *M. lutarioriparius* chromosomes were performed, respectively. The slides previously stained or hybridized were washed twice for 15 min each in 2 × SSC, dehydrated through an ethanol series (70%, 90% and 100%, 5 min each) and then used for hybridization. The *in situ* hybridization and detection were performed as described by She et al. (2006). Briefly, 40 μl of the hybridization mixture, which contained 20 μl 20% dextran sulfate, 1 μl ssDNA, 16 μl hybridization buffer (HB50, containing 50% deionized formamide and 50 mM sodium phosphate, pH 7.5) and 3 μl labeled DNA (final concentration 100–150 ng/slide), was added to each slide and covered with a 24 × 50 mm glass coverslip. Chromosomes and probe were denatured together on ThermoBrite S500-24 (Abbott Molecular, USA) at 80 °C for 3 min, and then were incubated at 37 °C for 24 h. Post-hybridization washing was performed in 0.1 × SSC two times for 15 min each at 42 °C, followed by rinsing in 2 × SSC three times for 5 min each at 42 °C and in TN buffer (containing 100 mM Tris-HCl and 150 mM NaCl, pH 7.5) for 5 min at room temperature. Hybridization signals were detected after incubating the slides with 100 μl TNB buffer (0.5% Roche blocking reagent in TN buffer) for 30 min at 37 °C, and followed by rinsing in TN buffer for 1 min at room temperature.

The biotin-labeled 45S rDNA was detected using Fluorescein Avidin D (Vector Laboratories). The digoxigenin-labeled gDNA was detected using Anti-digoxigenin-rhodamine (Roche Diagnostics). The specific steps were as follows: 100 μl of 1% Fluorescein Avidin D or Anti-digoxigenin-rhodamine, diluted with TNB buffer, was added to each slide and covered with a glass coverslip, and then were incubated at 37 °C in dark for 1 h. Afterwards, the coverslip was removed and rinsed with TN buffer three times for 5 min each in dark. Slides were counterstained with 3 µg/ml DAPI in a 30% solution of Vectashield H100 and subsequently examined under an epifluorescence microscope equipped with the CCD camera as mentioned above. Observations were made using a UV, blue and green excitation filters for DAPI, fluorescein, and rhodamine, respectively. Grey-scale images were digitally captured and merged by the Ocular software. The final images were adjusted with Adobe PHOTOSHOP CS8.0.

### Karyotype analysis

For each species, five well-spread metaphase plates were measured using Adobe PHOTOSHOP CS8.0 to obtain the chromosome relative lengths (RL; % of haploid complement), arm ratios (AR = long arm/short arm), chromosome length ratio (longest chromosome length / shortest chromosome length), size of the fluorochrome band (expressed as a percentage of the karyotype length) and the percentage distance from the rDNA site to the centromere (di = d × 100/a; d = distance of the centre of the rDNA sites from the centromere; a = length of the corresponding chromosome arm) (Greilhuber and Speta 1976). In addition, the total length of the haploid complement (TCL; i.e. karyotype length) was measured using the five metaphase cells with the highest degree of chromosome condensation. The arm ratio was used to classify the chromosomes according to the system described by Levan et al. (1964). Karyotype asymmetry was determined using the mean centromeric index (CI), the intrachromosomal asymmetry index (A1), the interchromosomal asymmetry index (A2) (Zarco 1986), the ratio of the length of all long arms in the chromosome set to the total chromosome length in set (As K%) (Arano 1963), the asymmetry index (AI) (Paszko 2006), and the categories of Stebbins (Stebbins 1971). The chromosomes were arranged in order of decreasing lengths. Idiograms were drawn based on chromosomes measurement data, fluorochrome bands and 45S rDNA FISH signals.

### The PCR and sequencing

The rDNA-ITS regions (including ITS1, 5.8s and ITS2) of the four *Miscanthus* species were amplified using the universal primers ITS4 and ITS5 (ITS4 primer sequence: 5’-TCCTCCGCTTATTGATATGC-3’, ITS5 primer sequence: 5’-GGAAGTAAAAGTCGTAACAAGG-3’) (White et al. 1990). The total volume of the PCR amplification reaction was 25 μl, including 2.5 μl 10 × PCR buffer, 1.5 μl 25 mM MgCl_2_, 0.5 μl 10 mM dNTP, 0.75 μl 10 μM of each primer, 0.5 μl Taq DNA polymerase (Sangon, Shanghai, China), 1.5 μl gDNA (30~50 ng/μl) and 17 μl ddH_2_O. The amplification conditions were: pre-denatured at 95 °C for 4 min; denaturation at 94 °C for 45 s, annealing at 54 °C for 45 s, extension at 72 °C for 45 s, 38 cycles; and a final extension step of 10 min at 72 °C on a thermal cycler PTC-200. The PCR products were detected by 1% agarose gel electrophoresis. PCR products purification and ITS sequencing were performed by Sangon. The ITS sequences have been deposited in GenBank and the accession numbers are listed in Table 1.

### DNA sequences and phylogenetic analyses

Each DNA sequence was spliced by bi-directional sequencing. Then, the similarity searches were performed using the NCBI (National Centre for Biotechnology Information), BLAST network service. Sequences were aligned with CLUSTAL W program. The MEGA 7.0 software (Kumar et al. 2016) was used for sequence analyses (estimating percentage of the G + C content, variable sites and parsimony informative sites). ITS sequences of *I. cylindrical* was used as the outer group plant, and phylogenetic analyses were carried out using the neighbour joining (NJ) and maximum parsimony (MP) methods. In the ITS phylogenetic tree, the confidence of each branch was tested using bootstrap (Felsenstein 1985), each performing 1 000 cycles to evaluate the systematic significance and reliability of each branch.

## Results

### Comparative karyotyping

**Figure 1.**
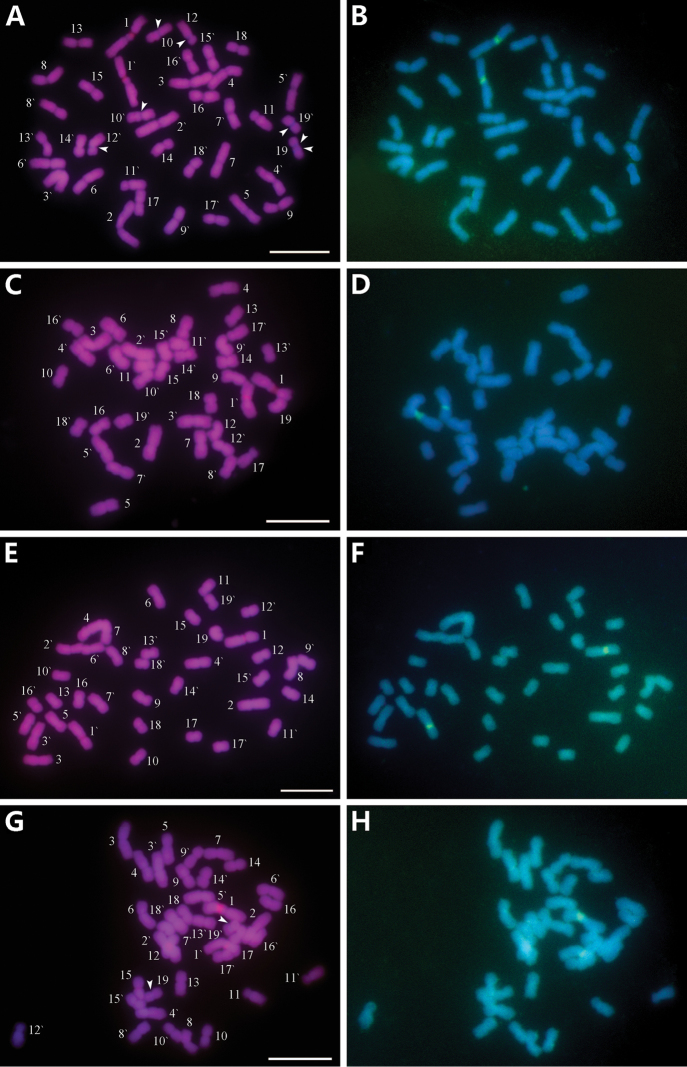
Mitotic chromosomes from *M. sinensis* (**A, B**), *M. sacchariflorus* (**C, D**), *M. lutarioriparius* (**E, F**) *M. floridulus* (**G, H**), stained with CPD staining and sequentially FISH with biotin-labelled 45S rDNA probe. **A, C, E** and **G** are chromosomes stained using CPD. The chromosome numbers were designated by karyotyping **B, D, F** and **H** are the chromosomes showing the 45S (green) signals. Arrowheads in **A** and **G** indicate the blue DAPI bands. Scale bars: 10 μm.

**Figure 2.**
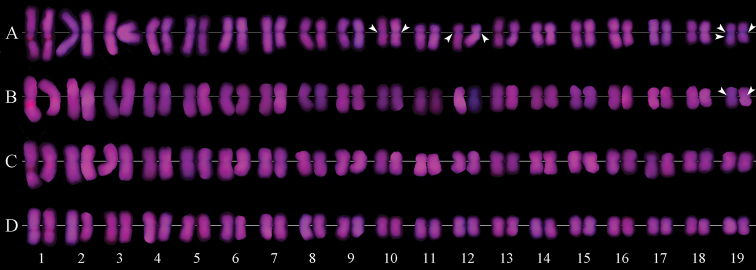
Karyotype showing CPD and DAPI bands of *M. sinensis* (**A**), *M. floridulus* (**B**), *M. sacchariflorus* (**C**), *M. lutarioriparius* (**D**). Arrowheads in **A** and **B** indicate the blue DAPI bands.

**Figure 3.**
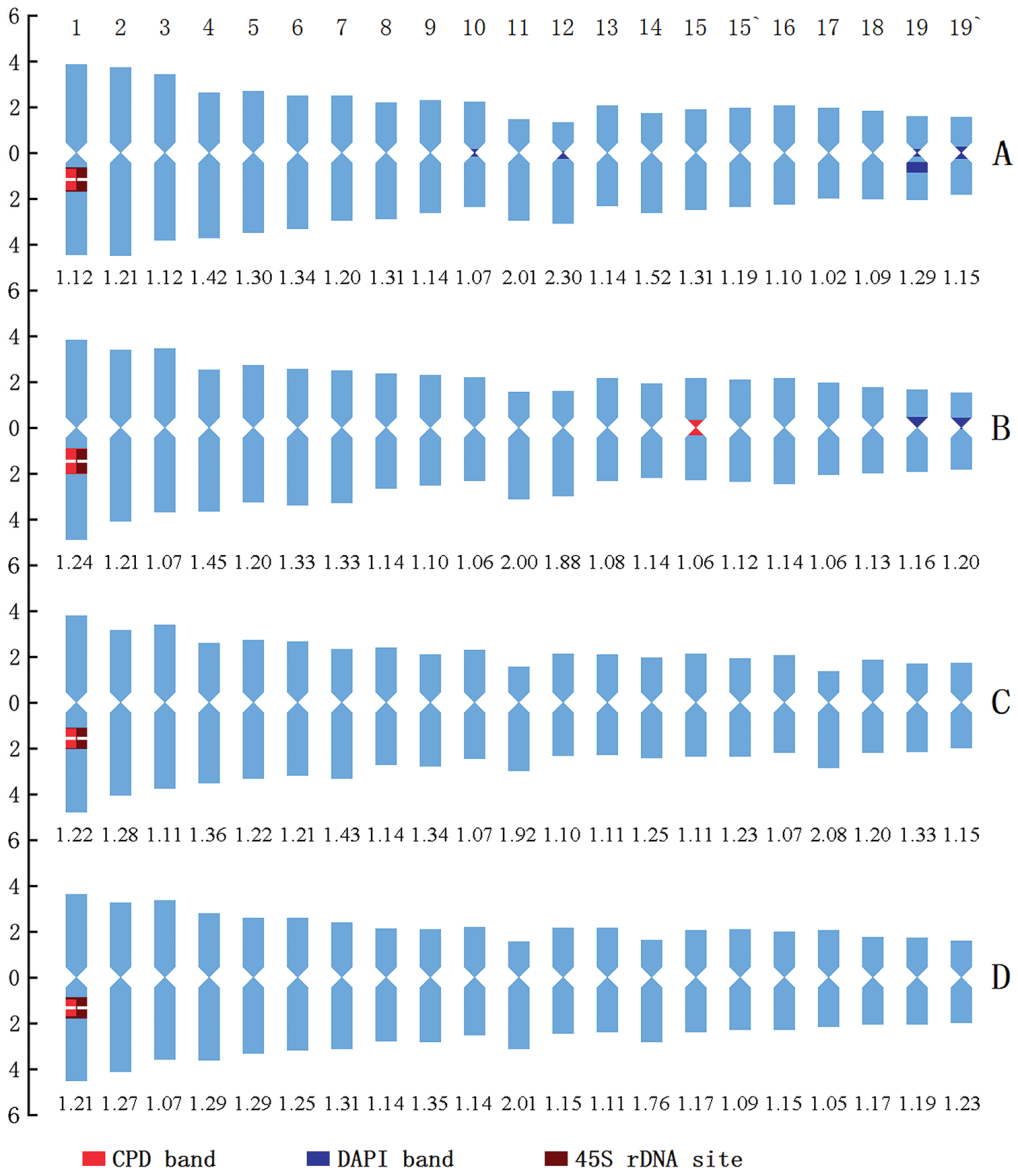
Idiograms of the four *Miscanthus* species that display the chromosome measurements, and the position and size of fluorochrome bands and 45 rDNA FISH signals. **A, B, C, D** indicate *M. sinensis*, *M. floridulus*, *M. sacchariflorus* and *M. lutarioriparius*, respectively. The ordinate scale on the left indicates the relative length of the chromosomes (i.e.% of haploid complement). The numerical values under each chromosome pair indicate the arm ratios of the respective chromosome pair. The numbers above panel **A** are chromosome numbers.

The karyotype measurement data for the four *Miscanthus* species are listed in Suppl. material 1, Tables S1–S4. The general karyotype features and parameters for the four *Miscanthus* species are listed in Table 2. Representative mitotic chromosomes, karyotypes showing the fluorescent bands and ideograms are shown in Figs 1–3.

**Table 2.** Karyotypic parameters of four *Miscanthus* species.

Species	KF	TCL±SD (μm)	RRL	CI±SD	A1	A2	As K%	AI	Stebbin’s types
*M. sinensis*	2n=2x=38=34m(2SAT)+4sm	73.92±2.87	3.53~8.23	44.00±4.97	0.20	0.27	55.85	3.06	2B
*M. floridulus*	2n=2x=38=34m(2SAT)+4sm	86.13±5.87	3.47~8.60	44.88±4.35	0.13	0.26	55.19	2.51	2B
*M. saccharifloru*s	2n=2x=38=34m(2SAT)+4sm	68.15±3.25	3.76~8.44	44.19±4.31	0.12	0.24	55.72	2.37	2B
*M. lutarioripariu*s	2n=2x=38=34m(2SAT)+4sm	76.48±5.02	3.69~8.04	44.56±3.83	0.11	0.52	55.40	4.47	2B

KF, karyotype formula; TCL, total length of the haploid complement (i.e. karyotype length); RRL, ranges of chromosome relative length; CI, mean centromeric index; A1 and A2, intra-chromosomal asymmetry index and inter-chromosomal asymmetry index, respectively; As K%, ratio of the length of all long arms in chromosome set to total chromosome length; AI, karyotype asymmetry index.

All the four *Miscanthus* species had diploid chromosome number of 2n = 2x = 38. The mitotic metaphase chromosomes with a mean chromosome length 3.59 μm for *M. sacchariflorus* and 4.53 μm for *M. floridulus*. The total length of the haploid complement (TCL) ranges from 68.15 μm to 86.13 μm, and the mean centromeric index (CI) of the complements varied slightly between 44.00 ± 4.97 and 44.81 ± 4.28. In contrast, *M. floridulus* has exhibited the large variation in chromosome length, whereas *M. sinensis* has displayed the large variation in centromeric index.

The karyotype formulas of the four *Miscanthus* species were same, composed of 34 metacentric (m) chromosomes and 4 submetacentric (sm) chromosomes with a secondary constriction located on the long arms of chromosome 1, namely 2n = 2x = 38 = 34m(2SAT) + 4sm. All the karyotypes of the four species studied fell into the categories 2B of Stebbins (1971). The ranges for Romero Zarco’s (1986) asymmetry indices were as follows: A1 = 0.11–0.20 and A2 = 0.24–0.52. The As K% of Arano (1963) ranged from 55.25 to 55.85, and Paszko’s (2006) asymmetry index (AI) ranged from 2.37 to 4.47. RRL, CI, A1 and As K% have shown close similarity among species. In contrast, TCL, A2, and AI have displayed relatively large variation among species. According to the AI values, the karyotype of *M. sacchariflorus* is the most symmetrical and that of *M. lutarioriparius* is the most asymmetrical among the four species.

### Fluorescence banding patterns

After CPD staining, slightly different fluorochrome banding patterns were observed among the four *Miscanthus* species (Fig. 1–3; Table 3). The red CPD bands were recorded in all species, whereas blue DAPI bands were found only in *M. sinensis* and *M. floridulus*.

**Table 3.** The distribution of fluorochrome bands and 45S rDNA sites in the four *Miscanthus* species.

Species	Fluorochrome bands			Number (pair) and location of 45S rDNA sites^†§^
	Type	Distribution^†^	amount^‡^ (%)	
*M. sinensis*	CPD	45S sites	0.93	One [1L-PROX (25.53%)]
	DAPI	10 CENS, 12L-PCENS, 19 CENS, 19L-PROX (one homologue)	1.94	
*M. floridulus*	CPD	45S sites, 15 PCEN (one homologue)	1.11	One [1L-PROX (29.43%)]
	DAPI	19S-PCEN	0.45	
*M. sacchariflorus*	CPD	45S sites	0.90	One [1L-PROX (32.07%)]
*M. lutarioriparius*	CPD	45S sites	0.75	One [1L-PROX (28.45%)]

^†^ S and L represent the short and long arms, respectively; CEN, PCEN and PROX represent the centromeric, pericentromeric and proximal positions, respectively; figures ahead of the positions designate the chromosomal pair involved. ^‡^ Amount of bands in the genome, expressed as a percentage of the karyotype length. ^§^ The percentages in parenthesis indicate the percentage distance from the centromere to the rDNA site (di = d × 100/a; d = distance of the centre of the 45S sites from the centromere, a = length of the corresponding chromosome arm).

Results showed that only one pair of CPD bands in *M. sinensis*, *M. sacchariflorus* and *M. lutarioriparius* had occurred in the secondary constrictions on the long arms of chromosome 1, and were co-localized with the 45S rDNA-FISH hybridization sites (called rDNA CPD bands; Fig. 1A, E, G). There were three CPD bands in the *M. floridulus*: two bands correspond to the secondary constriction on the long arms of chromosome 1; the other band was a non-rDNA CPD band with weaker fluorescence, occurring in the pericentromeric region of a homologue of chromosome pair 15 (Fig. 1G). The rDNA CPD bands in *M. sinensis* and *M. lutarioriparius* were similar in size and intensity on the two homologous chromosomes, while those in *M. floridulus* and *M. sacchariflorus* displayed heterozygosity, the band on one chromosome was large and bright, whereas the band on the other homologue was small and weak. The CPD bands of *M. sinensis*, *M. floridulus*, *M. sacchariflorus* and *M. lutarioriparius* accounted for 0.93%, 1.11%, 0.90% and 0.75% of the karyotype length, respectively.

*M. sinensis* showed seven blue DAPI bands (Fig. 1A, 2A, 3A): two pairs of weak bands occurred in the centromeric regions of chromosomes 10 and the pericentromeric regions of the long arm of chromosome 12, three relatively strong bands occured on chromosome 19. Among the DAPI bands on pair 19, two were located in the centromeric regions of both homologues, and one occured in the proximal region of the long arm of one homologue. *M. floridulus* had shown only one pair of weak DAPI bands in the pericentromeric regions on the chromosomes 19 (Fig. 1G, 2B, 3B). The DAPI bands of *M. sinensis* and *M. floridulus* accounted for 1.94% and 0.45% of the karyotype length, respectively.

### FISH mapping of 45S rDNA

45S rDNA FISH showed that *M. sinensis*, *M. floridulus*, *M. sacchariflorus* and *M. lutarioriparius* had only one pair of 45S rDNA sites, which were located in the secondary constriction on the long arms of chromosome 1, and their percentage distances of 45S rDNA sites were 25.53 ± 1.17, 29.43 ± 1.12, 32.07 ± 0.49, 28.45 ± 0.89, respectively. The 45S rDNA sites of the four *Miscanthus* species corresponded to their respective CPD bands in both size and intensity, that is, the 45S rDNA signals of two homologues in *M. sinensis* and *M. lutarioriparius* were similar in size and intensity, while those in *M. floridulus* and *M. sacchariflorus* differed in size and intensity, displaying heterozygosity.

### GISH signal patterns

**Figure 4.**
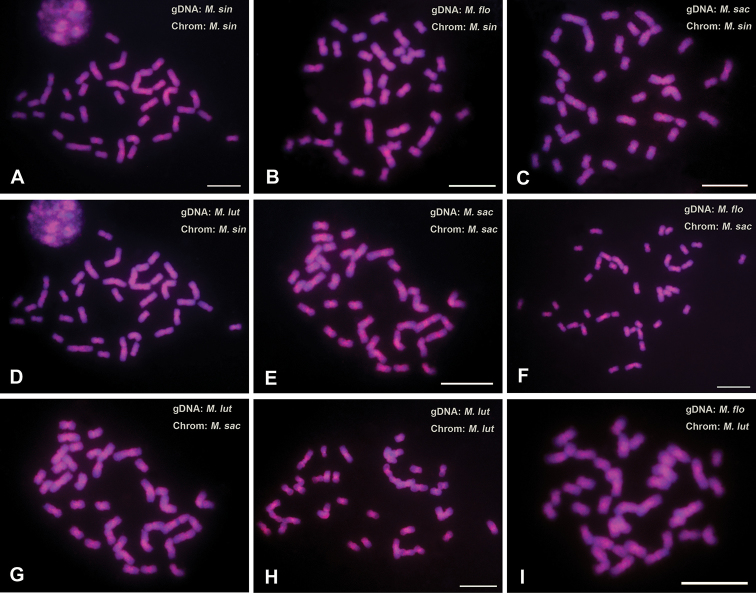
FISH with *M. sinensis* (**A**), *M. floridulus* (**B**), *M. sacchariflorus* (**C**) and *M. lutarioriparius* (**D**) genomic DNA probes (red) to *M. sinensis* chromosomes, FISH with *M. sacchariflorus* (**E**), *M. floridulus* (**F**) and *M. lutarioriparius* (**G**) genomic DNA probes (red) to *M. sacchariflorus* chromosomes, and FISH with *M. lutarioriparius* (**H)** and *M. floridulus* (**I**) genomic DNA probes (red) to *M. lutarioriparius* chromosomes. Scale bars: 10 μm.

The GISH results are shown in Fig. 4. Both self-GISH (sGISH; the genomic DNA of a species is applied to its own chromosomes) and cGISH generated hybridization signals in most regions of all chromosomes. Overall, the hybridization signals in the proximal and/or centromeric regions of the chromosomes were strong or very strong, while those in the proximal regions were relatively weak. In the GISHs to the *M. sinensis* chromosomes, the signals generated by *M. floridulus*gDNA (Fig. 4B) were stronger and more evenly distributed than those generated by *M. sacchariflorus* and *M. lutarioriparius* gDNAs (Fig. 4C, D), and more similar to the sGISH signals of *M. sinensis* (Fig. 4A). The hybridization signals of *M. floridulus*gDNA to the chromosomes of *M. sacchariflorus* (Fig. 4F) and *M. lutarioriparius* (Fig. 4I) were weaker than both the sGISH signals of *M. sacchariflorus* (Fig. 4E) and *M. lutarioriparius* (Fig. 4H), and the cGISH signals of *M. lutarioriparius*gDNA to *M. sacchariflorus* chromosomes (Fig. 4G) .

### Phylogeny analysis based on ITS

Each ITS1-5.8S-ITS2 sequences were compared to the published sequences of *Miscanthus* and its related species, and the boundaries of the spacer regions were confirmed. The length and other characteristics of each ITS1-5.8S-ITS2 are given in Table 4. The entire ITS sequence (ITS1-5.8S-ITS2) of *I. cylindrica* that was used as the outgroup species was 684 bp in length, and its GC content was 63.89%.

**Table 4.** Features of the ITS1-5.8S-ITS2 sequences of the four *Miscanthus* species.

	Length range	G/C content range (%)	No. of indels	No. of variable sites	No. of informative sites	Transitions	Transversions	Ratio
ITS1	258–260	67.44–68.46	2	7	5	2	3	2:3
5.8S	157	56.05–56.69	0	1	0	0	1	0:1
ITS2	244–245	60.25 –61.63	1	8	3	1	6	1:6
complete	659–661	62.37–63.09	3	16	8	3	10	3:10

**Figure 5.**
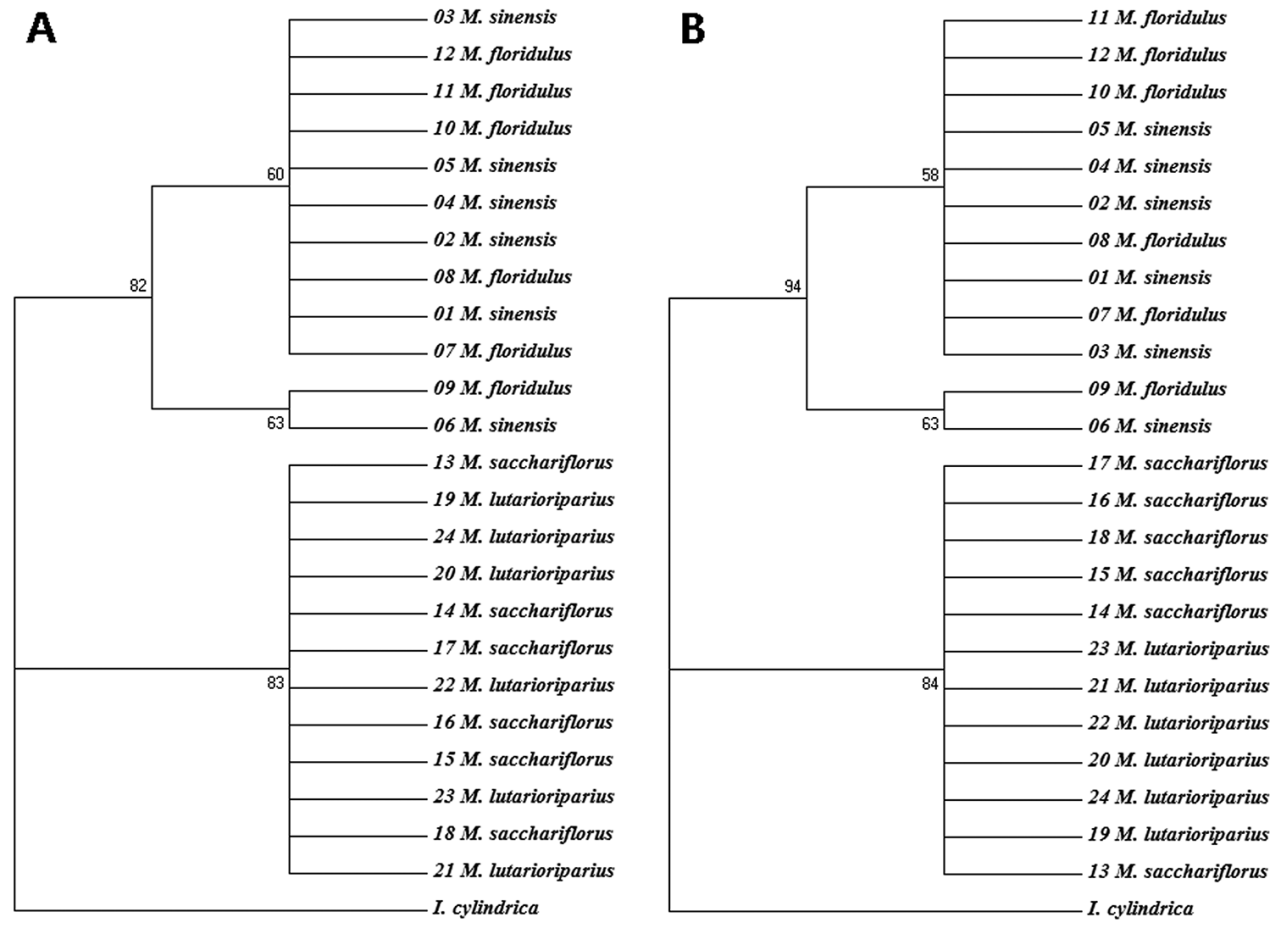
Rooted neighbour-joining (NJ) (**A**) and maximum-likelihood (ML) (**B**) tree based on the ITS1-5.8S-ITS2 sequences of the four *Miscanthus* species using the MEGA software (version 7.0) with *I. cylindrica* as an outgroup species. The numbers near the nodes indicate bootstrap values (in percentage).

Neighbour joining (NJ) and maximum likelihood (ML) phylogenetic trees were developed based on the entire ITS sequences. The NJ and ML trees were very similar (Fig. 5), and the four *Miscanthus* species were divided into two categories: (i) group I contained *M. sinensis* and *M. floridulus*, which resulted in 82% (NJ) and 94% (ML) bootstrap values; (ii) group II included *M. sacchariflorus* and *M. lutarioriparius*, with 83% and 84% bootstrap values in NJ and ML trees, respectively. It was worth noting that in each branch the accessions of one species were not separated from those of another species (Fig. 5).

## Discussion

### Molecular cytogenetic karyotypes

In the present study, detailed karyotypes of *M. sinensis*, *M. floridulus*, *M. sacchariflorus* and *M. lutarioriparius* were established using a combination of chromosome measurements, fluorochrome bands and 45S rDNA FISH signals, which provided the primary molecular-cytogenetic characterization of the four *Miscanthus* energy plants for the first time.

Our results had shown that the molecular cytogenetic karyotypes were rather similar among the four *Miscanthus* species. For instance, their karyotype formula, the categories of Stebbins, and the number and location of the 45S rDNA sites were same and there were slight differences in RRL, CI, A1 and As K%. However, several differences in their karyotypes were recorded: (1) obvious differences in their TCL, A2 and AI. In particular, AI, which can more accurately reveal the heterogeneity of chromosome length and centromere index in karyotype (Paszko 2006), had significant differences among the four species. (2) A non-rDNA CPD band appeared in *M. floridulus*. (3) AT-rich heterochromatin (DAPI bands) occurred in *M. sinensis* and *M. floridulus* but not in *M. sacchariflorus* and *M. lutarioriparius*. (4) There appeared 45S rDNA heterozygosity in *M. floridulus* and *M. sacchariflorus* but not in *M. sinensis* and *M. lutarioriparius*. Therefore, the four *Miscanthus* species could be accurately distinguished from each other using the molecular-cytogenetic karyotypic data.

As our study revealed, the chromosome numbers of the four *Miscanthus* species were all 2n = 2× = 38, being consistent with those reported previously (Hodkinson et al. 2002a, Takahashi and Shibata 2002, Takahashi et al. 2002, Chramiec-Głąbik et al. 2012). The current karyotypes of the four *Miscanthus* species comprised mainly of metacentric chromosomes, differing from the previous karyotypes, which had more submetacentric chromosomes, and even had acroentric chromosomes (Chramiec-Głąbik et al. 2012). Our study revealed that each species had a pair of satellite chromosomes, which were designated as chromosome 1. Previous studies also reported the presence of satellite chromosomes in *Miscanthus* species and revealed by FISH one pair of 45S rDNA sites in *M. sinensis*, but the serial number of the satellite chromosomes and the locations of the secondary constrictions were different (Takahashi and Shibata 2002, Takahashi et al. 2002, Chramiec-Głąbik et al. 2012). The deviations in karyotype were probably mainly due to differences in the material analysed, and difficulty in accurately pairing homologous chromosomes and distinguishing chromosomes by using the classical staining technique as applied before.

### Phylogenetic relationships of the four *Miscanthus* species

The research on the evolutionary relationship among the four *Miscanthus* species could not be conducted using genomic information because the genome sequences of *M. floridulus*, *M. sacchariflorus* and *M. lutarioriparius* were currently unavailable. Therefore, at present the combination of molecular cytogenetic analysis with rDNA ITS and chloroplast DNA sequence analysis was an effective phylogenetic analysis pathway (She et al. 2015).

As mentioned above, the molecular cytogenetic karyotypes of the four *Miscanthus* species were very similar, indicating the high genome similarity and small genome differentiation among them. Furthemore, the cGISH signals were rather similar in intensity and distribution to the sGISH signals, further demonstrating the high similarity among the four *Miscanthus* genomes (Wolny and Hasterok 2009, She et al. 2015, Zhang et al. 2015). However, the differences in molecular cytogenetic karyotypes and cGISH signals among the four species also provided valuable evolution information: (1) AT-rich heterochromatin appeared in both *M. sinensis* and *M. floridulus*, but did not emerge in both *M. sacchariflorus* and *M. lutarioriparius*; (2) The hybridization signals of *M. floridulus*gDNA to *M. sinensis* chromosomes were stronger and more evenly distributed than those of *M. sacchariflorus* and *M. lutarioriparius*gDNA, whereas the hybridization signals of *M. floridulus*gDNA to *M. sacchariflorus* chromosomes were weaker than the hybridization signals of *M. lutarioriparius*gDNA to *M. sacchariflorus* chromosomes. This information indicates that there was a close phylogenetic relationship between *M. sinensis* and *M. floridulus*, and between *M. sacchariflorus* and *M. lutarioriparius*; the former two species were relatively distant from the latter two species.

In our study, the phylogenetic tree based on the ITS sequences had shown that, *M. sinensis* and *M. floridulus* clustered into one branch, and *M. sacchariflorus* and *M. lutarioriparius* clustered into another branch. It was consistent with not only the above molecular cytogenetic results, but also the previous clustering results based on morphology, molecular markers and the ITS sequence (Hodkinson et al. 2002b, Chen et al. 2007, Sun et al. 2010, Chae et al. 2014, Clark et al. 2014, Ge et al. 2017). In addition, our clusering analysis revealed that the accessions of the two species in each branch were mixed and without distinct boundaries. These findings were consistent with the phylogenetic trees of *Miscanthus* and related genera inferred from ITS sequences (Hodkinson et al. 2002b, Chen et al. 2007, Liu et al. 2010), SSR markers (Ge et al. 2017), SNPs (Clark et al. 2014) and the dataset of genome size, ploidy level and genomic polymorphisms (Chae et al. 2014). To summarise, our clustering results have demonstrated that there was a very close phylogenetic relationship between *M. sinensis* and *M. floridulus*, and between *M. sacchariflorus* and *M. lutarioriparius* and they cannot be distinguished only based on the ITS sequences. However, as revealed in this study, the molecular cytogenetic karyotype analysis can effectively identify the four species.

### Conclusion

Molecular cytogenetic karyotypes of *M. sinensis*, *M. floridulus*, *M. sacchariflorus* and *M. lutarioriparius* were established for the first time, which can effectively distinguish the four species. Molecular cytogenetic comparison revealed basic similarities and certain differences in genome organization among the four species. These findings will provide a reference for further development of specific probes based on *M. sinensis* genome sequence for chromosomal localization in the species of *Miscanthus* and related genera. The combined data of molecular cytogenetic and ITS sequence analysis indicated a close phylogenetic relationship between *M. sinensis* and *M. floridulus*, and between *M. sacchariflorus* and *M. lutarioriparius*, respectively. It can be concluded that former two species have relatively distant relationship compared with the latter two species.

## Supplementary materials

Supplementary material 1 Molecular cytogenetic characterization and phylogenetic analysis of four *Miscanthus* species (Poaceae) Data type: species data Explanation note: **Table S1.** Chromosome measurements of *M. sinensis*. **Table S2**. Chromosome measurements of *M. floridulus*. **Table S3.** Chromosome measurements of *M. sacchariflorus*. **Table S4.** Chromosome measurements of *M. lutarioriparius*. https://binary.pensoft.net/file/324637This dataset is made available under the Open Database License (http://opendatacommons.org/licenses/odbl/1.0/). The Open Database License (ODbL) is a license agreement intended to allow users to freely share, modify, and use this Dataset while maintaining this same freedom for others, provided that the original source and author(s) are credited.Yanmei Tang, Liang Xiao, Yasir Iqbal, Jianfeng Liao, Longqian Xiao, Zili Yi, Chaowen She
