# Supplementary material for: Molecular cytogenetic characterization and phylogenetic analysis of four Miscanthus species (Poaceae)
Source: Comp Cytogenet. 2019 Aug 9;13(3):211–30. doi: 10.3897/CompCytogen.v13i3.35346 (PMC6697684; doi:10.3897/CompCytogen.v13i3.35346)
Supplement: Supplementary material 1 [file comparative_cytogenetics-13-211-s001.docx]

**Table 1.** Chromosome measurements of *M. sinensis*

| **Chromosome**  **No.** | **Relative length (%)** | | | **Arm ratio±SD** | **Type** |
| --- | --- | --- | --- | --- | --- |
|  | **short arm±SD** | **long arm±SD** | **total±SD** |  |  |
| 1 | 3.88±0.15 | 4.35±0.20 | 8.23±0.35 | 1.12±0.01 | m |
| 2 | 3.73±0.26 | 4.50±0.01 | 8.22±0.27 | 1.21±0.08 | m |
| 3 | 3.44±0.24 | 3.84±0.09 | 7.27±0.33 | 1.12±0.06 | m |
| 4 | 2.64±0.10 | 3.75±0.19 | 6.38±0.29 | 1.42±0.02 | m |
| 5 | 2.70±0.05 | 3.51±0.06 | 6.21±0.08 | 1.30±0.03 | m |
| 6 | 2.49±0.08 | 3.35±0.05 | 5.84±0.14 | 1.34±0.02 | m |
| 7 | 2.49±0.08 | 2.98±0.30 | 5.47±0.32 | 1.20±0.12 | m |
| 8 | 2.21±0.10 | 2.90±0.08 | 5.11±0.16 | 1.31±0.05 | m |
| 9 | 2.30±0.04 | 2.62±0.14 | 4.93±0.17 | 1.14±0.05 | m |
| 10 | 2.22±0.03 | 2.38±0.02 | 4.60±0.05 | 1.07±0.00 | m |
| 11^†^ | 1.48±0.11 | 2.97±0.12 | 4.45±0.14 | 2.01±0.17 | sm |
| 12^†^ | 1.35±0.14 | 3.08±0.21 | 4.43±0.33 | 2.30±0.16 | sm |
| 13 | 2.07±0.21 | 2.34±0.05 | 4.42±0.21 | 1.14±0.13 | m |
| 14 | 1.74±0.36 | 2.62±0.39 | 4.36±0.72 | 1.52±0.19 | m |
| 15 | 1.94±0.06 | 2.42±0.07 | 4.36±0.11 | 1.25±0.03 | m |
| 16 | 2.06±0.14 | 2.27±0.16 | 4.33±0.28 | 1.10±0.04 | m |
| 17 | 1.97±0.10 | 2.01±0.13 | 3.98±0.24 | 1.02±0.02 | m |
| 18 | 1.85±0.05 | 2.02±0.14 | 3.87±0.15 | 1.09±0.08 | m |
| 19 | 1.60±0.16 | 1.94±0.06 | 3.54±0.22 | 1.22±0.09 | m |

SD, standard deviation. m, metacentric chromosome. sm, submetacentric chromosome. ^†^ satellite chromosome, the length of the satellite was included in the chromosome length but that of the stretched secondary constriction was excluded.

**Table 2.** Chromosome measurements of *M. floridulus*

| **Chromosome**  **No.** | **Relative length (%)** | | | **Arm ratio±SD** | **Type** |
| --- | --- | --- | --- | --- | --- |
|  | **short arm±SD** | **long arm±SD** | **total±SD** |  |  |
| 1 | 3.85±0.18 | 4.75±0.50 | 8.60±0.40 | 1.24±0.18 | m |
| 2 | 3.39±0.10 | 4.10±0.04 | 7.49±0.09 | 1.21±0.04 | m |
| 3 | 3.46±0.03 | 3.70±0.19 | 7.16±0.20 | 1.07±0.05 | m |
| 4 | 2.53±0.08 | 3.65±0.00 | 6.18±0.08 | 1.45±0.04 | m |
| 5 | 2.73±0.06 | 3.27±0.12 | 6.01±0.08 | 1.20±0.07 | m |
| 6 | 2.56±0.00 | 3.41±0.11 | 5.97±0.12 | 1.33±0.04 | m |
| 7 | 2.49±0.06 | 3.31±0.07 | 5.80±0.13 | 1.33±0.01 | m |
| 8 | 2.37±0.19 | 2.68±0.11 | 5.06±0.11 | 1.14±0.14 | m |
| 9 | 2.30±0.05 | 2.53±0.15 | 4.83±0.13 | 1.10±0.08 | m |
| 10 | 2.21±0.08 | 2.35±0.11 | 4.56±0.19 | 1.06±0.02 | m |
| 11^†^ | 1.58±0.11 | 3.14±0.29 | 4.72±0.24 | 2.00±0.28 | sm |
| 12^†^ | 1.59±0.07 | 3.00±0.17 | 4.59±0.24 | 1.88±0.02 | sm |
| 13 | 2.17±0.17 | 2.34±0.19 | 4.51±0.36 | 1.08±0.02 | m |
| 14 | 1.94±0.09 | 2.20±0.04 | 4.14±0.13 | 1.14±0.04 | m |
| 15 | 2.14±0.13 | 2.33±0.11 | 4.47±0.23 | 1.09±0.04 | m |
| 16 | 2.16±0.07 | 2.46±0.19 | 4.63±0.24 | 1.13±0.06 | m |
| 17 | 1.96±0.07 | 2.07±0.13 | 4.03±0.17 | 1.06±0.06 | m |
| 18 | 1.78±0.08 | 2.01±0.08 | 3.79±0.03 | 1.13±0.09 | m |
| 19 | 1.60±0.11 | 1.88±0.12 | 3.48±0.11 | 1.19±0.14 | m |

SD, standard deviation. m, metacentric chromosome. sm, submetacentric chromosome. ^†^ satellite chromosome, the length of the satellite was included in the chromosome length but that of the stretched secondary constriction was excluded.

**Table 3.** Chromosome measurements of *M. sacchariflorus*

| **Chromosome**  **No.** | **Relative length (%)** | | | **Arm ratio±SD** | **Type** |
| --- | --- | --- | --- | --- | --- |
|  | **short arm±SD** | **long arm±SD** | **total±SD** |  |  |
| 1 | 3.80±0.15 | 4.65±0.17 | 8.44±0.25 | 1.22±0.06 | m |
| 2 | 3.17±0.04 | 4.07±0.16 | 7.25±0.18 | 1.28±0.05 | m |
| 3 | 3.40±0.15 | 3.76±0.22 | 7.15±0.36 | 1.11±0.02 | m |
| 4 | 2.60±0.11 | 3.53±0.09 | 6.13±0.03 | 1.36±0.09 | m |
| 5 | 2.75±0.10 | 3.34±0.01 | 6.09±0.11 | 1.22±0.04 | m |
| 6 | 2.65±0.05 | 3.21±0.06 | 5.87±0.03 | 1.21±0.04 | m |
| 7 | 2.35±0.02 | 3.35±0.15 | 5.70±0.16 | 1.43±0.06 | m |
| 8 | 2.41±0.04 | 2.74±0.19 | 5.15±0.22 | 1.14±0.07 | m |
| 9 | 2.10±0.06 | 2.81±0.03 | 4.91±0.09 | 1.34±0.03 | m |
| 10 | 2.29±0.05 | 2.47±0.04 | 4.76±0.07 | 1.07±0.02 | m |
| 11^†^ | 1.57±0.09 | 3.00±0.17 | 4.56±0.11 | 1.92±0.21 | sm |
| 12 | 2.14±0.13 | 2.34±0.23 | 4.48±0.27 | 1.10±0.12 | m |
| 13 | 2.10±0.21 | 2.31±0.05 | 4.41±0.26 | 1.11±0.09 | m |
| 14 | 1.95±0.13 | 2.44±0.03 | 4.40±0.10 | 1.25±0.09 | m |
| 15 | 2.03±0.14 | 2.37±0.07 | 4.40±0.10 | 1.17±0.11 | m |
| 16 | 2.06±0.12 | 2.20±0.16 | 4.26±0.27 | 1.07±0.03 | m |
| 17^†^ | 1.38±0.01 | 2.86±0.12 | 4.24±0.14 | 2.08±0.07 | sm |
| 18 | 1.85±0.22 | 2.20±0.10 | 4.05±0.12 | 1.20±0.19 | m |
| 19 | 1.68±0.09 | 2.08±0.02 | 3.76±0.07 | 1.24±0.08 | m |

SD, standard deviation. m, metacentric chromosome. sm, submetacentric chromosome. ^†^ satellite chromosome, the length of the satellite was included in the chromosome length but that of the stretched secondary constriction was excluded.

**Table 4.** Chromosome measurements of *M. lutarioriparius*

| **Chromosome**  **No.** | **Relative length (%)** | | | **Arm ratio±SD** | **Type** |
| --- | --- | --- | --- | --- | --- |
|  | **short arm±SD** | **long arm±SD** | **total±SD** |  |  |
| 1 | 3.64±0.18 | 4.40±0.15 | 8.04±0.16 | 1.21±0.09 | m |
| 2 | 3.26±0.13 | 4.13±0.24 | 7.38±0.18 | 1.27±0.11 | m |
| 3 | 3.37±0.08 | 3.60±0.20 | 6.98±0.24 | 1.07±0.05 | m |
| 4 | 2.81±0.16 | 3.63±0.20 | 6.44±0.33 | 1.29±0.06 | m |
| 5 | 2.61±0.28 | 3.34±0.11 | 5.95±0.19 | 1.29±0.17 | m |
| 6 | 2.59±0.24 | 3.20±0.19 | 5.80±0.14 | 1.25±0.19 | m |
| 7 | 2.41±0.20 | 3.15±0.05 | 5.56±0.22 | 1.31±0.11 | m |
| 8 | 2.47±0.17 | 2.81±0.04 | 5.28±0.16 | 1.14±0.08 | m |
| 9 | 2.10±0.01 | 2.83±0.19 | 4.93±0.19 | 1.35±0.09 | m |
| 10 | 2.21±0.05 | 2.53±0.06 | 4.74±0.03 | 1.14±0.05 | m |
| 11^†^ | 1.58±0.15 | 3.14±0.21 | 4.72±0.23 | 2.01±0.26 | sm |
| 12 | 2.16±0.14 | 2.47±0.03 | 4.63±0.11 | 1.15±0.09 | m |
| 13 | 2.16±0.10 | 2.40±0.03 | 4.56±0.08 | 1.11±0.07 | m |
| 14^†^ | 1.62±0.15 | 2.83±0.04 | 4.45±0.12 | 1.76±0.17 | sm |
| 15 | 2.08±0.03 | 2.35±0.11 | 4.43±0.10 | 1.13±0.07 | m |
| 16 | 2.01±0.07 | 2.31±0.12 | 4.32±0.15 | 1.15±0.07 | m |
| 17 | 2.07±0.10 | 2.17±0.04 | 4.24±0.11 | 1.05±0.06 | m |
| 18 | 1.78±0.05 | 2.08±0.12 | 3.86±0.16 | 1.17±0.05 | m |
| 19 | 1.67±0.12 | 2.02±0.08 | 3.69±0.13 | 1.21±0.10 | m |

SD, standard deviation. m, metacentric chromosome. sm, submetacentric chromosome. ^†^ satellite chromosome, the length of the satellite was included in the chromosome length but that of the stretched secondary constriction was excluded.
